# Effectiveness of a Virtual Reality Immersion Process as a Nonpharmacological Intervention for Behavioral and Psychological Symptoms of Dementia and Immediate Well-Being in People Living in Nursing Homes: Randomized Controlled Trial

**DOI:** 10.2196/95542

**Published:** 2026-10-06

**Authors:** Marion Giroux, Floriane Delphin-Combe, Marianne Rion, Mohamed Nour Temedda, Virginie Dauphinot, Stéphanie Tripoz-Dit-Masson, Clémence Grangé, Antoine Garnier-Crussard, Sophie Dautricourt

**Affiliations:** 1 Hospices Civils de Lyon, Clinical and Research Memory Centre of Lyon, Lyon Institute for Aging, Hôpital des Charpennes 27 Rue Gabriel Péri, Villeurbanne, Rhône-Alpes, 69100, France, 33 0472432174; 2 INSERM, LIMICS, Sorbonne Université, Paris; 3 Emeis Group Research Department, Emeis group, Puteaux, France; 4 CNRS, Inserm, centre de recherche en neurosciences de Lyon CRNL, Université Claude-Bernard - Lyon 1, Bron, France

**Keywords:** neuropsychiatric symptoms, behavioral and psychological symptoms of dementia, Alzheimer disease, nonpharmacological interventions, nursing home, virtual reality, short-term well-being

## Abstract

**Background:**

Behavioral and psychological symptoms of dementia (BPSD) are common among nursing home residents, notably those with Alzheimer disease and related diseases, impacting both themselves and health care professionals. Nonpharmacological interventions, including virtual reality (VR), have shown promise, but evidence from multicenter ecological studies remains limited.

**Objective:**

This study aimed to evaluate the effectiveness, in terms of BPSD evolution, immediate well-being, and social interactions, of a VR-based immersion intervention (Lumeen) as a nonpharmacological approach to managing BPSD among nursing home residents. It also aimed to confirm the feasibility of implementing and evaluating group-based immersive VR interventions in nursing homes.

**Methods:**

Fifty-six nursing home residents with BPSD took part in this multicenter randomized controlled trial (ClinicalTrials.gov NCT04769024), receiving either VR-based sessions or an active control condition consisting of nondigital cognitive stimulation. Both interventions consisted of two 45-minute group sessions per week for 6 weeks, resulting in a total of 12 sessions in each intervention group. BPSD severity was assessed preintervention and postintervention using the Neuropsychiatric Inventory (NPI) and the Apathy Interview. Immediate well-being and social interaction were assessed at each session using the Immediate Well-Being Evaluation and the Social Observation Behaviors Residents Index, respectively.

**Results:**

No significant between-group differences were found in BPSD reduction or in overall well-being outcomes. However, immediate well-being increased from presession to postsession in both groups when averaged across the 12 sessions, and social interactions were more frequent in the active control group. Only 2 mild adverse events were reported.

**Conclusions:**

VR interventions in nursing home residents with neurocognitive disorders did not demonstrate superiority over an active cognitive stimulation control in reducing BPSD. Both interventions were associated with improvements in immediate well-being, suggesting that nonpharmacological group-based activities may be beneficial in nursing home settings. Additionally, VR interventions were safe and feasible in this population. Immersive VR warrants further investigation in adequately powered pragmatic trials to clarify its specific added value and optimal implementation conditions for BPSD.

## Introduction

In 2024, people over 65 represented 21.5% of the French population [1]. An aging population is associated with an increase in the prevalence of age-related diseases and, thus, an increase in the prevalence of neurocognitive disorders (NCDs). Indeed, NCD is a syndromic diagnosis defined by the *Diagnostic and Statistical Manual of Mental Disorders* (*DSM*) and could be due to several underlying diseases, such as Alzheimer disease, dementia with Lewy bodies, or vascular dementia. The high prevalence of major NCDs (ie, dementia), along with the significant loss of autonomy and quality of life they entail, makes them a major public health issue.

In addition to cognitive impairment, behavioral and psychological symptoms of dementia (BPSD), also called neuropsychiatric symptoms, are common in NCDs. There is a wide range of BPSD, from apathy to agitation, including, for example, depression, irritability, and anxiety [2]. The presence of BPSD is associated with increased disability, reduced quality of life [3] for both patients and caregivers [4], higher mortality risk [5], and a greater likelihood of nursing home admission for individuals with major NCDs [6,7]. BPSD are, therefore, highly prevalent in nursing homes, with reported rates reaching up to 96% [2].

Since disease-modifying therapies are currently unavailable for most NCDs, symptomatic management remains a cornerstone of care, particularly for BPSD, to improve the quality of life for both individuals with NCDs and their caregivers. Nonpharmacological interventions are considered first-line treatments for BPSD [8-10], as they have fewer side effects and are at least as effective as pharmacological treatments (ie, psychotropic drugs) [11,12], which are frequently prescribed in nursing homes [13]. In recent decades, nonpharmacological interventions leveraging the latest digital technologies have been developed, with virtual reality (VR) emerging as a promising approach. VR allows participants to be immersed either in 360° real-world videos or in computer-generated environments, and initial findings suggest beneficial effects. Among the various VR interventions available, passive immersive experiences depicting natural and culturally meaningful environments have attracted growing interest. Such environments may promote relaxation, engagement, reminiscence, and social interaction while requiring minimal cognitive or motor demands, making them particularly suitable for people living with NCDs [14-17].

First, VR is well-accepted by older adults [18] as well as by individuals with major NCDs [19,20]. For example, a study using a VR headset to present short 360° video clips during hospitalization in acute care found that immersive VR was well-tolerated by people with major NCDs and led to enjoyment and relaxation [20].

Additionally, a meta-analysis indicated that VR interventions could have significant effects on various domains, including physical fitness, cognition, and emotional well-being in individuals with NCDs [21]. When specifically examining the impact of VR on BPSD, several studies have reported positive outcomes. For instance, a randomized controlled trial including 69 people with major NCDs hospitalized in an acute care found that participants in the VR group, who watched short 360° videos using a VR headset, exhibited reduced aggression compared with those receiving standard care [22].

Some studies have explored VR interventions in individual settings [22-26], while others have adopted a group-based approach [23,27]. Regardless of the intervention format, studies have reported reductions in apathy [24], agitation [25], and depressive symptoms [26], although these effects were often observed only during VR sessions. For example, a study found that a single immersive VR session showing relaxing scenes—delivered either individually or in groups—led to a reduction in apathy in 13 nursing home residents with major NCDs [23]. Another study involving 25 nursing home residents, 64% of whom had cognitive impairment, showed that 6 immersive VR group sessions resulted in decreased depression beyond the sessions, while increased pleasure, greater alertness, and reduced apathy were observed only during immersion [27].

While these findings are promising, the current literature exhibits several methodological limitations that warrant caution. As noted in a recent review, many studies have not been conducted in ecological settings, such as nursing homes, which limits the generalizability of results to clinical practice. For example, only 1 out of 19 studies identified by Appel et al [17] was conducted in a nursing home [17]. Furthermore, a significant number of studies exhibit limitations in experimental design; very few have used a valid comparison group, and sample sizes were often small. For example, 17 out of 19 studies identified by Appel et al had fewer than 40 participants, with 8 having 10 or fewer participants [17]. Additionally, there is substantial heterogeneity in the technology used, with some studies using partially immersive interfaces (eg, 2D screens) rather than head-mounted displays [24], which may not provide comparable effects.

The aim of this multicenter randomized controlled trial was to evaluate the effectiveness of a group-based, fully immersive VR intervention in reducing BPSD in ecological settings (ie, several nursing homes), with a valid comparison group (ie, a group-based activity commonly used in nursing homes: nondigital cognitive stimulation), and a moderately larger sample size. In addition to its clinical outcomes, this study also offers information regarding the feasibility of implementing and evaluating group-based immersive VR interventions in nursing homes. We hypothesized that VR group interventions would lead to a greater reduction in BPSD compared to group-based nondigital cognitive stimulation, particularly in reducing apathy.

## Methods

### Participants

The study was conducted between December 2022 and February 2024 in 5 nursing homes located in the Auvergne-Rhône-Alpes region of France. Two facilities were situated in Lyon, accommodating 80 and 103 residents, respectively. A third facility, located in a village on the outskirts of Lyon, housed 80 residents. The remaining 2 facilities were in Le Puy-en-Velay, with capacities of 45 and 49 residents. Nursing homes were initially selected from the Korian private group, which had granted study authorization, and recruitment was later extended to facilities located near Lyon and Le Puy-en-Velay to increase the number of participating centers. Participants included in the study lived in one of these 5 nursing homes and had BPSD, measured by the Neuropsychiatric Inventory (NPI) [28,29], with a score ≥9 on at least 1 of the following symptoms: apathy, agitation, anxiety, depression, or aberrant motor behaviors, since the VR intervention was developed to focus mainly on these symptoms. An NPI threshold of ≥9 was used to identify symptoms considered both severe and frequent. Exclusion criteria were BPSD severity preventing group participation, infectious neurological diseases, substance abuse, nonstabilized psychiatric disorders, severe uncompensated sensory deficits, epilepsy, visual hallucinations, pacemakers, severe or uncontrolled heart conditions, scalp burns, and scalp sores. Based on the recruitment capacity and the feasibility of carrying out the interventions in the different centers, it was planned to recruit 12 participants in each center (ie, a planned sample size of 60 participants). Randomization was stratified by center and was conducted electronically (Ennov Clinical) by the investigator, with a maximum of 6 participants per group per center. Resident capacities for each center, as well as the numbers of participants enrolled and included in the final analyses, are presented in Multimedia Appendix 1. The random allocation sequence was accessible only to the data manager and was concealed from personnel responsible for participant enrollment and assignment. Given the nature of the intervention, blinding of participants and intervention facilitators was not feasible.

### Ethical Considerations

This study (IDRCB 2021-A00297-34-A) was approved by the local ethics committee (Comité de Protection des personnes EST I, France) on June 30, 2022, and was registered on ClinicalTrials.gov with the title “Virtual Reality Intervention for the Reduction of Behavioral and Psychological Symptoms of Dementia (LUMEENCOMPAD)” with registration number NCT04769024. Participants provided written informed consent, or, if unable, their relative or legal guardian did so, with the participant’s oral consent still considered. Individuals unable to consent and without a relative or legal guardian were excluded.

### Outcomes

The primary outcome was the NPI, a French version (entitled NPI-ES for “*équipes soignantes*”), the equivalent of the NPI for nursing homes [28,29]. Responses to the NPI were collected from health care staff by a trained health care professional (ie, psychologist or medical doctor). The NPI was designed to assess changes in the patient’s behavior by gathering information from someone who knows the patient well (ie, a family or professional caregiver), enabling clinical follow-up and treatment evaluation. It assesses 12 BPSD (delusions; hallucinations; agitation and aggression; depression and dysphoria; anxiety; elation and euphoria; apathy and indifference; disinhibition; irritability and lability; aberrant motor behavior; nighttime behavioral disturbances; and appetite and eating abnormalities) in terms of frequency (range 0‐4) and severity (range 0‐3). The score is calculated by multiplying the frequency and severity for each dimension and adding up each of these subscores (range 0-144; 144 being the maximum score).

Secondary outcomes were immediate well-being, social interactions, and apathy symptoms.

Immediate well-being was assessed using the Immediate Well-Being Evaluation (IWBE), a visual analog scale developed for people with major NCD, even at a severe stage (eg, Mini-Mental State Examination [MMSE] <10) [30]. Participants had to answer the question “How are you feeling right now?” by moving a slider on the scale. On the side presented to the participant, the scale shows pictograms of simple facial expressions (ie, joy, neutral, and sadness) and it goes from “Not Well” (on the left) to “Very Well” (on the right). On the other side, facing the evaluator, the position of the slider is associated with numbers from 0 (the lowest feeling of well-being) to 5 (the highest); Multimedia Appendix 2.

Social interactions were assessed by the Social Observation Behaviors Residents Index (SOBRI), an observation grid evaluating several interaction behaviors (eg, “mutual gaze” and “nodding”) toward peers and health care professionals [31] during the interaction time. It is composed of 16 items, which can be coded as 0 or 1 depending on their occurrence (0 indicates absence; 1 indicates presence of the behavior). Thus, the total score ranges from 0 to 16. An external observer, a clinical research coordinator, completed the SOBRI during each of the 12 sessions, observing each participant separately for 4 minutes.

Apathy symptoms were assessed using the Apathy Interview [32], a scale proposed to health care staff to evaluate 3 symptoms of apathy (ie, emotional blunting, lack of initiative, and loss of interest). For each symptom, its intensity is estimated from 0 (absence of the symptom) to 4 (major). The total score ranges from 0 (absence of apathy) to 12 (major apathy).

Adverse events were systematically recorded after each session, and their seriousness was assessed by the investigator.

### Procedure

#### Study Design

This open-label, multicenter randomized controlled trial had 2 parallel arms. Due to staff and room constraints, centers could not run both groups simultaneously. Therefore, an alternating implementation schedule was adopted across centers. Centers were enrolled sequentially according to the chronological date of study implementation, with the first center conducting the control group first, the second the intervention group first, and so forth. This resulted in 3 centers implementing the control group first and 2 centers implementing the intervention group first. This procedure was introduced for logistical purposes only; participants were individually assigned to a single study condition and were not exposed to both conditions. The study outline for each implementation schedule is shown in Figure 1.

**Figure 1.**
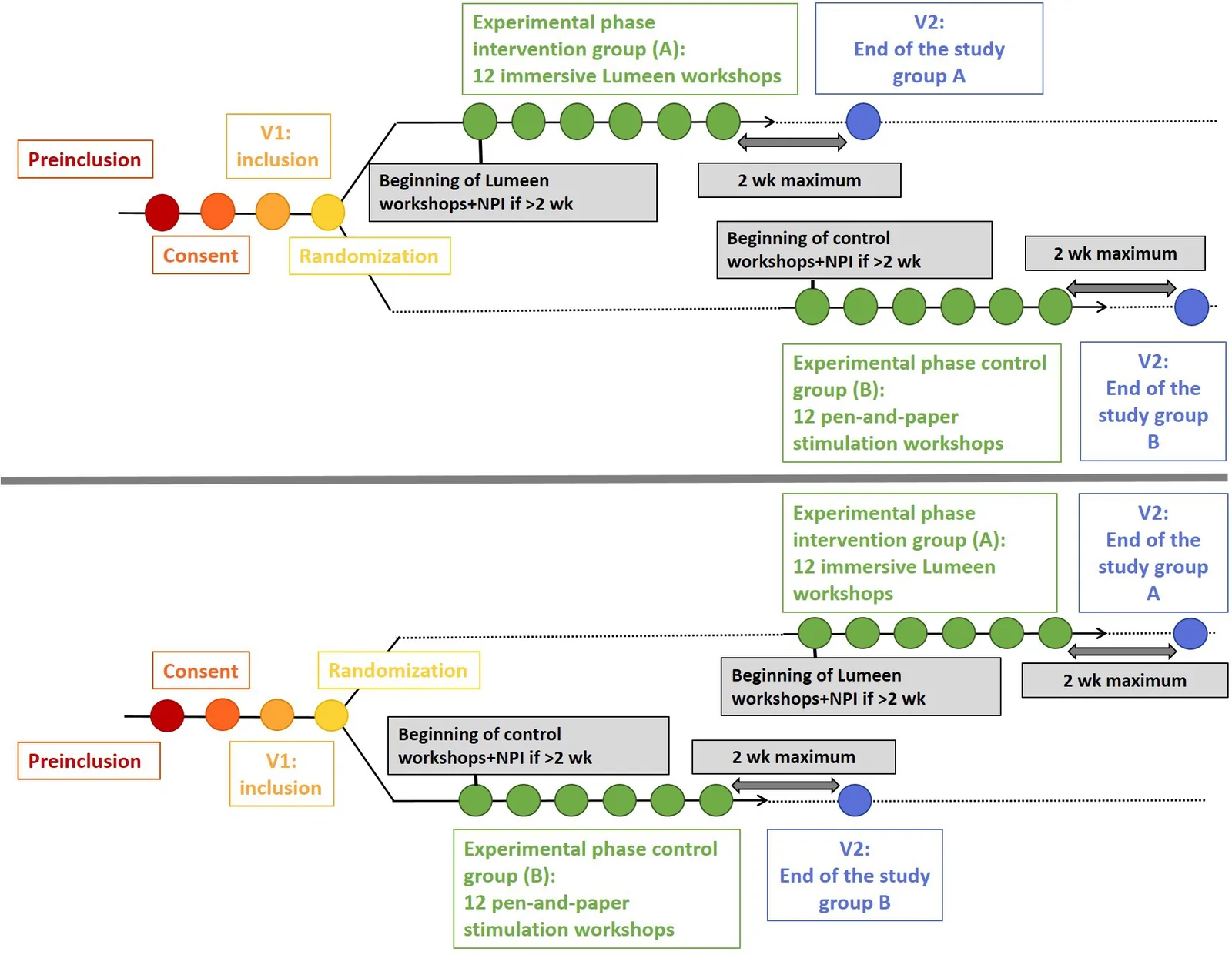
General outline of the study for centers starting with the intervention group (group A) in the upper panel, and general outline of the study for centers starting with the control group (group B) in the lower panel.

#### Preinclusion and Consent

A screening of the medical record was performed by the investigator (ie, the coordinating physician of the nursing home) to identify potential participants. The investigator then presented the study to the residents and their relatives or legal representatives and collected consent.

#### Inclusion (V1) and Randomization

During this phase, the investigator rechecked the inclusion and exclusion criteria. If the last NPI was over 2 weeks old, a new one was conducted. The Apathy Interview was rated, and the MMSE was performed when possible, unless one had been done in the past 3 months.

#### Interventions

##### Intervention Delivery and Session Procedures

Both the intervention and control groups were composed of a maximum of 6 participants per center. There were two 45-minute group sessions per week for 6 weeks (12 sessions in total) in each group. Facilitators were members of the health care staff (eg, psychologist and professional entertainer) who had been previously trained in the VR intervention and in the nondigital cognitive stimulation intervention. An observer completed the observation grid of social interaction (SOBRI) in each session and measured IWBE before and after each session.

##### Intervention Group: VR (Lumeen)

Each center used Lumeen software (version 2.8.1; Lumeen SAS), preinstalled on a digital tablet, along with 6 associated VR headsets with 3 df (Pico G2 4K). Lumeen is Class I medical device software, Conformité Européenne marked and identified by its Basic Unique Device Identifier–Device Identifier 3770025309LUMEEN-V2F7. The Evasion module developed by Lumeen provides standardized 360° live-action videos of natural and cultural environments with ambient sounds and music. This type of passive immersive content has been reported to be feasible and well tolerated in people with NCDs and may promote well-being and engagement while requiring no active navigation or complex user interactions [14,16,33].

Each video lasts between 7 and 12 minutes and is presented with appropriate mediation content. The content was synchronized across the 6 headsets so that participants saw the same 360° video at the same time. Table 1 lists the videos presented in this study with their descriptions, and Figure 2 shows pictures from these videos.

**Table 1.** List of the virtual reality videos used, in this order, in the intervention group of the study.

Title	Description
Animals from around the world	A journey to discover animals: kangaroos, deer, ponies, etc
The Norwegian nature	Take a journey through the Norwegian landscape through the 4 seasons and discover Viking culture
At the heart of traditional Japan	Journey through Japan: traditional village, rice fields, and shrines among cherry blossom
The salty lands of “La Baie, de Somme”	Views of “La Baie de Somme,” contemplation of the sea, and observation of the sheep in the salt meadows
Alaska under the Northern Lights	A journey through incredibly colorful landscapes and the Northern Lights
A walk in Lyon	A walk through the most emblematic sites in the city of Lyon
A dive with the dolphins	Underwater diving up close to the dolphins
The Somme and its farmers.	A walk in the fields with farmers, driving a tractor, milking cows, and making cheese
The Bouglione Winter Circus 2	Circus show excerpt: hoop act, clown, and acrobat
Walk in the Cévènes Ardéchoises	Panoramic views of the countryside, medieval village, and canoeing along the Ardèche river
The Pyrenees on board the Train Jaune	Journey on board a tourist train in Occitania
The goats of the Ciran farm	Visit a goat’s cheese farm: caring for the animals, milking, and making the cheese

**Figure 2.**
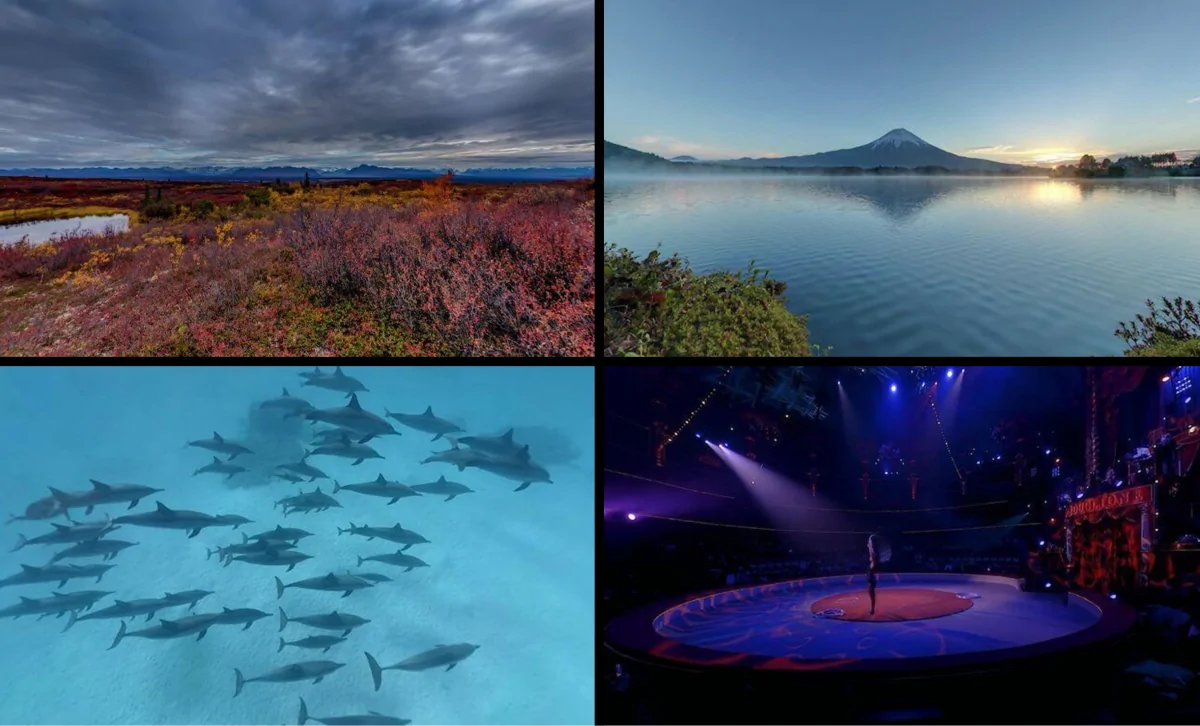
Pictures from the 3D virtual reality videos used in the study: Alaska under the Northern Lights (top left), in the heart of traditional Japan (top right), diving with dolphins (bottom left), and Bouglione Winter Circus 2 (bottom right).

Participants were seated in armchairs in a clear environment with enough space between them so they could move their arms. The facilitator introduced the content of the session (5 min). The participants were then fitted with VR headsets to see the content planned for the session (Table 1) for 7 to 12 minutes. The headsets were taken off, and the facilitator led an interaction time (25 min). During the interaction period, the facilitator used a standardized set of questions displayed on the tablet controlling the headsets to encourage participants to share their impressions of the VR content. The same questions were used across all participating centers. It was during this interaction time that the external observer completed the SOBRI, based on 4 predefined consecutive minutes of observation for each participant.

##### Active Control Group: Pen-and-Paper Activities

A panel of nondigital activities for cognitive stimulation, as well as instructions for their use, was provided to each center. These were pen-and-paper activities usually offered to people with cognitive disorders in nursing homes and were divided into four categories; see Figure 3 for examples:

1. Interaction: Media to interact with participants (eg, a list of things, such as movies, for which participants have to say whether they know and like each item).
2. Language: Tasks using verbal material (eg, sorting words, completing sentences by the more logical word, anagrams, and crosswords).
3. Semantic memory: General knowledge and reasoning (eg, quizzes or sentences to sort according to whether they relate to the present or the past).
4. Visual: Tasks using visual abilities (eg, “spot the differences” games, finding the odd image out, and coloring).

**Figure 3.**
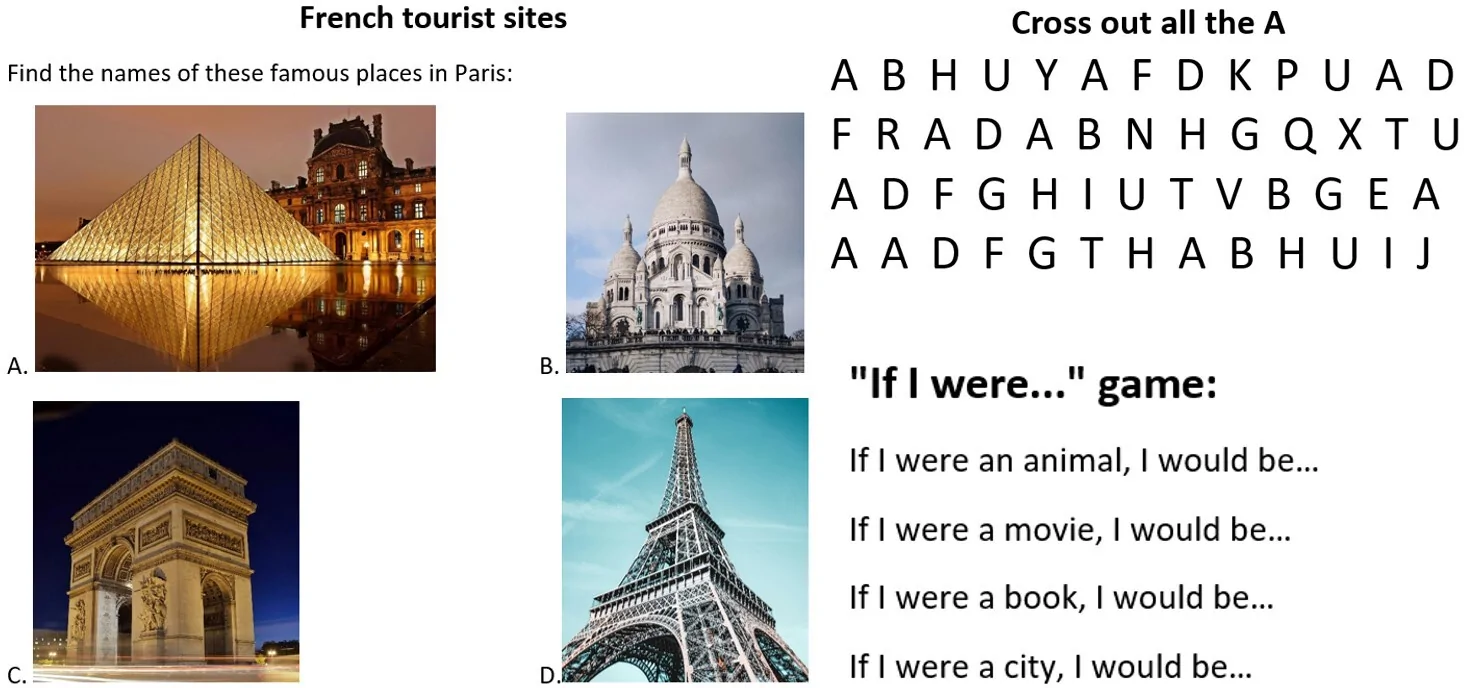
Examples of nondigital activities provided for the control group, translated into English.

Participants sat at a table while the facilitator provided various activities. An example program was given to guide facilitators of the control groups, but they could choose activities from the panel provided (see above) based on participants’ preferences and abilities, as in typical cognitive stimulation groups. During the 45-minute session, they were encouraged to include activities from different categories while prioritizing participants’ pace over strict adherence to a program.

All nursing homes provided additional activities alongside our intervention. Participation in these activities was not systematically monitored; however, staff ensured that residents did not engage in other VR-based or digital interventions during the study period.

### End of the Study (V2)

The last evaluation of the participants was conducted within 2 weeks following the final session, depending on caregiver and facility availability. The NPI and the Apathy Interview were rated, and a questionnaire was completed to report any major event in the life of the resident that could interact with the results of the study (eg, death of a loved one or deterioration in general condition). The reporting of this randomized controlled trial followed the CONSORT (Consolidated Standards of Reporting Trials) 2025 statement (Checklist 1).

### Covariates

Sociodemographic characteristics (ie, age and sex) were extracted from participants’ medical records by the investigator at inclusion. The MMSE was administered at baseline whenever feasible; otherwise, the most recent score recorded within the preceding 3 months was retrieved from the medical record. As intervention and control participants were equally distributed within each center, any center-specific effects were naturally balanced between groups. Consequently, center was not included as a covariate in the analyses.

### Statistical Analysis

Participant characteristics and various measures were compared between the intervention and control groups using the Chi-square/Fisher test for proportions and 2-tailed Student’s *t* test for means (SDs). For scores assessed preintervention and postintervention, mean (SD) values of all preintervention and postintervention scores were calculated. The effect of the intervention on the outcomes (ie, NPI, SOBRI, IWBE, and Apathy Interview) was evaluated using generalized linear mixed models with group, time, and the interaction between time and group as fixed factors, and a random intercept for participants. The models were adjusted for age, sex, and number of sessions to account for differences in intervention exposure across participants due to participants missing 1 or more sessions. Additional analyses, including MMSE as an adjustment variable, were conducted but could not be included in the primary analyses due to the number of missing MMSE data. Results were presented as standardized β coefficients (effect sizes) for each fixed effect, the standard error, and the *P* value. For the analyses, participants were excluded if they attended only 1 or no session, or if preintervention and/or postintervention measurements were missing.

All tests were 2-tailed, and a *P* value of less than .05 was considered statistically significant. For each analysis, the number of participants analyzed is indicated in brackets. This number depends on the presence of missing data. Analyses were performed using SPSS software (version 20.0; IBM Corp) and R software (version 4.1.2; R Foundation for Statistical Computing).

## Results

### Demographic Data and Baseline Scores

Fifty-six participants were enrolled, but 3 were excluded for not attending any sessions, leaving 53 participants (27 intervention and 26 control). The mean age was 85.7  (SD 8.3) years, with 60.4% (32/53) aged 85 or older. Most were women (42/53, 79.3%), and Alzheimer disease was the most common diagnosis (30/53, 56.6%). No baseline differences were found between groups in demographics or assessment measures (NPI, Apathy Interview, IWBE, and SOBRI); Table 2. A distribution of participants with each NPI symptom by group (intervention/control) and study phase (preintervention/postintervention), according to frequency and severity levels, is also provided in Multimedia Appendix 3.

**Table 2.** Demographic characteristics of the population and assessment criteria at baseline^a^.

Demographic characteristics and assessment criteria	Total (N=53)	Intervention (n=27)	Control (n=26)	*P* value
Age (y), mean (SD)	85.74 (8.28)	84.89 (8.59)	86.62 (8.04)	.45
Women, n (%)	42 (79.25)	22 (81.48)	20 (76.92)	.94
Marital status, n (%)				.91
Single	6 (11.32)	3 (11.11)	3 (11.54)	
Married	11 (20.75)	7 (25.93)	4 (15.38)	
Divorced	4 (7.55)	2 (7.41)	2 (7.69)	
Widowed	29 (54.72)	14 (51.85)	15 (57.69)	
Missing values	3 (5.66)	1 (3.70)	2 (7.69)	
MMSE^b^				.48
Mean (SD)	13.68 (4.69)	14.27 (4.82)	13.00 (4.64)	
Missing values^c^, n (%)	25 (47.17)	12 (44.44)	13 (50)	
NCD^d^ clinical diagnosis, n (%)				.85^e^
Alzheimer disease	30 (56.60)	15 (55.56)	15 (57.69)	
Other cause of NCDs (eg, Parkinson disease dementia)	15 (28.30)	9 (33.33)	6 (23.08)	
Unknown etiological diagnosis	8 (15.09)	3 (11.11)	5 (19.23)	
Number of sessions				.24
Mean (SD)	9.07 (3.03)	8.59 (3.45)	9.57 (2.48)	
Median (IQR)	10 (8‐12)	9 (6.5‐12)	10 (8‐11.75)	
NPI^f^, mean (SD)	28.26 (12.57)	27.26 (12.83)	29.31 (12.26)	.56
SOBRI^g^				
SOBRI-peers, mean (SD)	3.28 (2.68)	3.08 (2.41)	3.5 (2.96)	.57
Missing value, n (%)	1 (1.89)	1 (3.70)	0 (0)	—^h^
SOBRI-staff, mean (SD)	4.35 (1.62)	4.15 (1.74)	4.54 (1.50)	.40
Missing value, n (%)	1 (1.89)	1 (3.70)	0 (0)	—
SOBRI-total, mean (SD)	7.64 (3.86)	7.23 (3.7)	8.04 (4.04)	.46
Missing value, n (%)	1 (1.89)	1 (3.70)	0 (0)	—
IWBE^i^, mean (SD)	3.34 (1.37)	3.20 (1.54)	3.49 (1.17)	.45
Missing value, n (%)	3 (5.66)	1 (3.70)	2 (7.69)	—
Apathy Interview, mean (SD)				
Emotional blunting	1.87 (1.34)	1.78 (1.42)	1.96 (1.28)	.62
Lack of initiative	2.63 (1.49)	2.56 (1.57)	2.69 (1.44)	.74
Loss of interest	2.13 (1.58)	2.11 (1.55)	2.15 (1.64)	.92
Apathy Interview total	6.62 (4.10)	6.44 (4.28)	6.81 (3.99)	.75

a Comparisons of categorical variables between groups were made using Pearson chi-square test or Fisher test, while comparisons of quantitative variables between groups were made using 2-tailed Student *t* test.

b MMSE: Mini-Mental State Examination.

c Missing values were mainly due to the assessment being considered infeasible by the clinical staff, particularly for participants with advanced cognitive impairment.

d NCD: neurocognitive disorder.

e Fisher test.

f NPI: Neuropsychiatric Inventory.

g SOBRI: Social Observation Behaviors Residents Index.

h Not applicable.

i IWBE: Immediate Well-Being Evaluation.

### Feasibility and Safety Data

Of the 53 participants, only 15 attended all 12 sessions, but the majority (n=46, 86.8%) attended at least 6 sessions with a median of 10 (IQR 8‐12) sessions. No significant difference in the number of sessions attended was observed between the intervention (mean 8.59, SD 3.45) and control (mean 9.57, SD 2.48) groups (*P*=.24). The number of sessions attended was added as an adjustment variable in the models for the analyses, and a summary of attendance and reasons for nonattendance is provided in Multimedia Appendix 4. One participant was excluded from the analyses because he attended only 1 session, and postintervention assessments were missing for 2 participants because they discontinued the study early; see Multimedia Appendix 5 for an equivalent flow diagram adapted to our study. Thus, 50 participants were analyzed for the primary outcome (NPI).

Only 2 research-related adverse events, out of the 481 recorded sessions, were reported in the intervention group, corresponding to known symptoms of motion sickness and remaining at the lowest stage of severity. No research-related adverse events were recorded in the control group.

### Effect on BPSD (NPI)

A trend toward a reduction in BPSD was observed between the beginning and the end of the study, although this reduction was not significant (β=−1.148, *P*=.07, n=50). Additionally, this reduction did not depend on the group (intervention or control; β=−3.49, *P*=.32, n=50); see details in Tables 3 and 4.

**Table 3.** Preintervention and postintervention mean (SD) scores for the Neuropsychiatric Inventory (NPI) and Apathy Interview depending on the group (intervention or control).

Outcome measure	Intervention, mean (SD)		Control, mean (SD)	
Pre	Post	Pre	Post
NPI 12	27 (12)	22.9 (9.7)	29.4 (12.5)	27 (14.2)
Apathy Interview total	6.6 (4.2)	7.1 (4.1)	6.8 (4.1)	5.9 (4.2)

### Social Interaction (SOBRI)

Social interactions observed during the sessions tended to be lower in the intervention group than in the control group (β=−1.789, *P*=.07, n=52). Social interactions did not appear to change over the course of the sessions (β=−0.455, *P*=.16, n=52). After adjustment for MMSE, the association between group allocation and social interactions was significant (β=−3.805, *P*=.001, n=28 participants with available MMSE). In this adjusted model, higher MMSE scores were also independently associated with a greater number of social interactions during the sessions (β=0.325, *P*=.007, n=28).

### Well-Being (IWBE)

The analysis showed no significant effect of the VR intervention on the feeling of immediate well-being when compared with the control group (β=0.281, *P*=.22, n=50). However, the IWBE score increased significantly between the beginning and the end of the sessions, regardless of the group (β=0.363, *P*<.001, n=50). The number of intervention sessions attended was positively associated with well-being (β=0.101, *P*=.01, n=50; Tables 3 and 4).

**Table 4.** Multivariate analysis using mixed models of the association between groups and outcomes (NPI^a^, SOBRI^b^, IWBE^c^, and Apathy Interview)^d^.

Outcome, model, and variable	Standardized β coefficients	Standard error of the estimate	*P* value
NPI-model 1 (n=50)			
Intercept	43.512	20.262	.04
Age (continue)	−0.040	0.208	.85
Men	−1.986	4.260	.64
Number of sessions	−1.042	0.649	.12
Intervention group	−3.494	3.513	.32
Time	−1.148	0.629	.07
Intervention group × time	−0.474	0.893	.60
NPI-model 2 (n=27)			
Intercept	63.547	25.191	.02
Age (continue)	−0.184	0.246	.46
Men	−4.847	6.021	.43
MMSE^e^	−0.100	0.504	.85
Number of sessions	−1.797	1.106	.12
Intervention group	−1.521	4.618	.74
Time	−1.911	0.861	.04
Intervention group × time	−0.652	1.501	.67
SOBRI-model 1 (n=52)			
Intercept	5.524	5.484	.32
Age (continue)	0.014	0.057	.80
Men	−0.577	1.161	.62
Number of sessions	0.214	0.169	.21
Intervention group	−1.789	0.960	.07
Time	−0.455	0.323	.16
Intervention group × time	0.263	0.490	.59
SOBRI-model 2 (n=28)			
Intercept	5.819	5.536	.30
Age (continue)	−0.018	0.055	.74
Men	−0.039	1.289	.98
MMSE	0.325	0.111	.007
Number of sessions	0.245	0.210	.25
Intervention group	−3.805	1.078	.001
Time	−0.818	0.484	.09
Intervention group × time	0.960	0.669	.15
IWBE-model 1 (n=50)			
Intercept	1.081	1.254	.39
Age (continue)	0.010	0.012	.39
Men	0.204	0.263	.44
Number of sessions	0.101	0.039	.01
Intervention group	0.281	0.228	.22
Time	0.363	0.099	<.001
Intervention group × time	−0.245	0.138	.08
IWBE-model 2 (n=27)			
Intercept	−0.400	1.528	.80
Age (continue)	0.016	0.015	.30
Men	0.617	0.337	.08
MMSE	0.016	0.030	.59
Number of sessions	0.179	0.058	.006
Intervention group	0.187	0.300	.54
Time	0.319	0.142	.03
Intervention group × time	−0.209	0.188	.27
Apathy Interview–model 1 (n=50)			
Intercept	2.326	6.677	.73
Age (continue)	0.022	0.068	.75
Men	2.677	1.403	.06
Number of sessions	0.205	0.214	.34
Intervention group	0.038	1.177	.97
Time	−0.415	0.251	.11
Intervention group × time	0.648	0.357	.08
Apathy Interview–model 2 (n=27)			
Intercept	1.775	9.355	.85
Age (continue)	0.036	0.091	.70
Men	2.804	2.236	.22
MMSE	0.111	0.187	.56
Number of sessions	−0.206	0.411	.62
Intervention group	1.606	1.714	.36
Time	−0.337	0.317	.30
Intervention group × time	0.860	0.555	.13

a NPI: Neuropsychiatric Inventory.

b SOBRI: Social Observation Behaviors Residents Index.

c IWBE: Immediate Well-Being Evaluation.

d Both model 1 and model 2 included group, time, and the interaction between time and group as fixed factors, with a random intercept for participants. Both models were adjusted for age, sex, and number of sessions. Additionally, model 2 was further adjusted for the Mini-Mental State Examination score. The number of participants included in each analysis is indicated in brackets. Postintervention NPI and Apathy Interview values were missing for 3 participants*.*

e MMSE: Mini-Mental State Examination.

### Apathy (Apathy Interview)

Apathy symptoms, as assessed using the Apathy Interview, were not significantly affected by the sessions (β=−0.415, *P*=.11, n=50), and there was no significant difference between the intervention and control groups (β=0.038, *P*=.97, n=50), Tables 3 and 4.

## Discussion

### Effectiveness of VR Intervention for BPSD in Nursing Home Residents

The primary aim of this multicenter randomized controlled trial was to evaluate whether a group-based VR intervention reduced BPSD compared with an active cognitive stimulation control in nursing home residents. Contrary to our hypothesis, VR did not significantly improve BPSD, apathy, or immediate well-being compared with the active control. However, both interventions improved immediate well-being, and VR was found to be safe and feasible to implement in routine nursing home practice.

This study did not identify significant differences between the 2 groups in terms of BPSD or apathy, contrasting with previous findings suggesting that VR could reduce aggressiveness [22], apathy [23,27,34], and depression [27]. One explanation may be that many earlier studies lacked a comparison group [23,27] or used standard care as a control [22,34], whereas ours used an active control group with cognitive stimulation. Another possibility is that the NPI, although widely used, may be insufficiently sensitive to detect subtle short-term changes in BPSD following nonpharmacological interventions. Additionally, the postintervention assessment was conducted at varying time points within the prespecified 2-week follow-up period, which may have introduced additional variability in the outcome measures and reduced our ability to detect intervention effects.

Although not the primary objective of the study, a notable finding was that immediate well-being improved following each session, regardless of the intervention type. This aligns with prior research reporting increased short-term pleasure [24,35], and satisfaction [36] following similar interventions.

Some trends in the results warrant further exploration. BPSD tended to decrease after the interventions, regardless of the group. As Gueyraud [37] noted, two 45-minute weekly sessions represent less than 0.5% of a resident’s week, so their clinical impact may be modest. This raises the question of whether such interventions, even if effective, yield clinically meaningful benefits. Larger samples may be required to detect small but meaningful effects, and it could be relevant to use multiple outcome measures to assess BPSD reduction—for instance, by monitoring medication use and its longitudinal changes.

These observations could also suggest the potential need for longer interventions, which raises another important yet unresolved issue concerning the structure of VR interventions. In this study, our schedule—two 45-minute sessions per week for 6 weeks—mirrors the existing literature, in which VR sessions typically last around 27 minutes, with 2.8 sessions per week over 7.6 weeks [17]. However, many studies do not report all parameters (duration, frequency, and total length) [17]. In our sample, many participants missed sessions, with a median attendance of 10 (IQR 8‐12) sessions. Attendance variability may have affected outcomes, as improvements in well-being appeared to be associated with the number of sessions attended.

Cognitive ability could also play a role in the effectiveness of these interventions. Cognitive decline is known to be associated with increased BPSD [38], especially apathy [39,40] and reduced well-being [41,42] and may influence the ability to engage in social interactions [43]. In our study, greater cognitive impairment appeared to be linked to reduced social engagement. This observation is consistent with previous literature and suggests that the NCDs severity may moderate the effects of nonpharmacological interventions. However, missing MMSE data prevented a more precise analysis of this relationship. Future studies should further investigate this relationship.

### Limitations

As stated above, attendance variability may have affected our results since well-being appeared to be associated with the number of sessions attended. However, because the interpretation of this covariate was not prespecified, this finding should be considered exploratory. Moreover, attendance was not randomly assigned, and participants who attended more sessions may also have differed from those with lower attendance in terms of motivation, health status, and fatigue. Therefore, this exploratory association between session attendance and improvements in well-being should be interpreted cautiously and cannot be considered evidence of a causal relationship. Nevertheless, the improvement in well-being remained significant after adjustment for session attendance, suggesting that the observed benefits cannot be explained solely by differences in exposure to study sessions. Taken together, these findings suggest that differences in attendance may have contributed to the observed improvement in well-being but are unlikely to account for it entirely. Future studies should further investigate factors influencing adherence and their impact on intervention outcomes.

A further limitation concerns the assessment of cognitive impairment. Missing MMSE data prevented a precise analysis of the relationship between NCD severity and intervention outcomes. These missing data were mainly due to the assessment being considered infeasible by the clinical staff, particularly for participants with advanced cognitive impairment. Consequently, NCD severity could not be systematically accounted for in our analyses. Furthermore, in line with the pragmatic design of the study and the principle of data minimization required during the ethical approval process, detailed clinical information such as comorbidities and medication use was not collected. As a result, adjustment for these potential confounders was not possible, and residual confounding cannot be excluded. Future studies should consider alternative measures of NCD severity, such as the Clinical Dementia Rating, along with more comprehensive clinical characterization of participants.

Personal preferences may also moderate the effects of VR interventions [44]. However, the design and sample size of the present study do not allow us to explore this question. While individualized VR interventions might prove more effective [44], they are also more demanding in terms of staff time. We therefore believe that both individualized and group-based approaches may be complementary, offering nursing homes different options depending on their available resources.

Methodological limitations should be considered. Despite a larger sample than many previous studies, statistical power was limited, particularly due to missing data (eg, MMSE, IWBE, and SOBRI) and participants’ absenteeism. Attendance was influenced by various factors, including weather conditions, medical appointments, fatigue, lack of motivation, or logistical constraints, reflecting real-world challenges in nursing homes. This limitation in statistical power, combined with an imbalance across BPSD categories, did not allow us to examine potential differential effects of VR on specific NPI subitems, even though it would have been of interest. This could be an interesting avenue for future research.

Additionally, the choice of comparison group could be viewed as a limitation. First, the control group received nondigital cognitive stimulation, and it is possible that a generational effect may make older adults naturally more inclined toward traditional activities than VR. Second, this control group effectively enhanced well-being and social interaction. While this may have made it harder to detect the benefits of VR, it offers a meaningful comparison with an accessible, widely used intervention. Comparing the intervention to an active rather than passive control helps isolate its specific therapeutic effects. Ideally, including a passive control group would have allowed us to distinguish these effects from natural fluctuations in well-being and the impact of study participation. However, the active control group still provides valuable insights by assessing the intervention’s added value beyond general engagement effects and existing alternatives.

A further limitation is that the social interactions were assessed based on only 4 minutes of observation during the 25-minute interaction period. This relatively brief observation window may have limited the ability to capture the full range and variability of participants’ responses. Additionally, the absence of video recordings prevented subsequent detailed behavioral analyses and may have reduced the precision of the observational measures.

Another limitation lies in the difficulty of ensuring that participants remained focused on the VR content during the intervention. It would be valuable for future studies to include such a measure, for example, by using eye-tracking VR headsets.

### Implications and Future Directions

Nonpharmacological VR interventions for BPSD are still evolving, and many knowledge gaps remain in the literature [45]. Our study contributes to this field by providing data from a randomized controlled trial in multiple nursing homes using an active control group and existing institutional resources (health care professionals as facilitators, available rooms, and standard equipment). This demonstrates the feasibility (and safety) of implementing such an intervention in nursing homes. With 53 participants, our sample exceeds most prior studies. A recent review found that, of 19 VR studies, 89.5% (n=17) had ≤40 participants, 26.3% (n=5) were randomized controlled trials, 5.3% (n=1) took place in nursing homes, and 36.8% (n=7) involved health care professionals as facilitators [17]. Our study, therefore, provides valuable insights using a rigorous methodology while maintaining ecological validity in real-world nursing home settings.

Beyond its clinical findings, this study also highlights the methodological challenges of conducting pragmatic intervention trials in nursing home residents with NCDs. Missing clinical data, variable attendance, difficulties in administering standardized cognitive assessments, and the need to minimize participant burden reflect the realities of conducting research in routine nursing home care rather than issues specific to this study. These challenges should therefore be considered not only limitations but also important considerations for the design of future pragmatic trials. Achieving methodological rigor in this population requires balancing internal validity with ecological validity to ensure that interventions remain both scientifically evaluable and feasible in routine clinical practice. Future studies should consider assessment tools applicable across the full spectrum of NCD severity (eg, the Clinical Dementia Rating), standardize the timing of postintervention assessments whenever possible, systematically document intervention exposure and participant engagement, and collect key clinical characteristics, including comorbidities and medication use, while balancing scientific needs with ethical and regulatory requirements regarding data minimization. Beyond evaluating VR itself, this study provides methodological insights that may help improve the design and conduct of future pragmatic trials in nursing home settings.

### Conclusion

This study highlights the potential benefits of VR group interventions for individuals with BPSD, particularly in enhancing immediate well-being with minimal associated risks. However, rather than demonstrating superiority over conventional cognitive stimulation, our findings suggest that immersive VR represents a feasible and safe alternative that warrants further investigation through adequately powered pragmatic trials.

These findings suggest that immersive VR should not necessarily be viewed as a replacement for existing nonpharmacological approaches, but rather as an additional therapeutic option whose value may depend on individual characteristics and implementation strategies. Larger pragmatic trials are now needed to identify which residents are most likely to benefit and under which conditions VR can provide clinically meaningful advantages in routine nursing home care.

## Supplementary material

10.2196/95542 Multimedia Appendix 1 Resident capacity of each center and numbers of participants enrolled and included in the final analyses.

10.2196/95542 Multimedia Appendix 2 Immediate Well-Being Evaluation.

10.2196/95542 Multimedia Appendix 3 Distribution of patients for each Neuropsychiatric Inventory symptom by group (intervention/control) and study phase (preintervention/postintervention), according to frequency and severity levels.

10.2196/95542 Multimedia Appendix 4 Attendance and reasons for not attending for each session.

10.2196/95542 Multimedia Appendix 5 Flow diagram adapted to our study.

10.2196/95542 Checklist 1 CONSORT 2025 checklist.

## Abbreviations

BPSD: behavioral and psychological symptoms of dementia
CONSORT: Consolidated Standards of Reporting Trials
*DSM*: *Diagnostic and Statistical Manual of Mental Disorders*
IWBE: Immediate Well-Being Evaluation
MMSE: Mini-Mental State Examination
NCD: neurocognitive disorder
NPI: Neuropsychiatric Inventory
SOBRI: Social Observation Behaviors Residents Index
VR: virtual reality

## References

[1] (2024). Population par sexe et groupe d’âges: données annuelles 2026 [Article in French]. INSEE (Institut national de la statistique et des études économiques).

[2] Cerejeira J, Lagarto L, Mukaetova-Ladinska EB (2012). Behavioral and psychological symptoms of dementia. Front Neurol.

[3] Brodaty H, Burns K (2012). Nonpharmacological management of apathy in dementia: a systematic review. Am J Geriatr Psychiatry.

[4] Shin IS, Carter M, Masterman D, Fairbanks L, Cummings JL (2005). Neuropsychiatric symptoms and quality of life in Alzheimer disease. Am J Geriatr Psychiatry.

[5] Nijsten JMH, Leontjevas R, Pat-El R, Smalbrugge M, Koopmans RTCM, Gerritsen DL (2017). Apathy: risk factor for mortality in nursing home patients. J Am Geriatr Soc.

[6] Gaugler JE, Yu F, Krichbaum K, Wyman JF (2009). Predictors of nursing home admission for persons with dementia. Med Care.

[7] Wancata J, Windhaber J, Krautgartner M, Alexandrowicz R (2003). The consequences of non-cognitive symptoms of dementia in medical hospital departments. Int J Psychiatry Med.

[8] (2009). Maladie d’Alzheimer et maladies apparentées: prise en charge des troubles du comportement perturbateurs [Report in French]. https://www.has-sante.fr/upload/docs/application/pdf/2009-07/maladie_dalzheimer-troubles_du_comportement_perturbateurs-recommandations.pdf.

[9] Livingston G, Huntley J, Sommerlad A (2020). Dementia prevention, intervention, and care: 2020 report of the Lancet Commission. Lancet.

[10] (2024). Nouvelles recommandations pour la prise en soins des Symptômes Psychologiques et Comportementaux (SPC) dans les maladies neurocognitives [Report in French]. https://sfgg.org/media/2024/09/SFGG-def-2024.pdf.

[11] Gill SS, Bronskill SE, Normand SLT (2007). Antipsychotic drug use and mortality in older adults with dementia. Ann Intern Med.

[12] McDermott O, Charlesworth G, Hogervorst E (2019). Psychosocial interventions for people with dementia: a synthesis of systematic reviews. Aging Ment Health.

[13] Novais T, Garnier-Crussard A, Reallon E, Mouchoux C, Gervais F (2025). Assessing the prevalence of anticholinergic and sedative medications to avoid in older adults from the French Health Data System. J Am Geriatr Soc.

[14] Clemente D, Romano L, Zamboni E, Carrus G, Panno A (2023). Forest therapy using virtual reality in the older population: a systematic review. Front Psychol.

[15] Coelho T, Marques C, Moreira D (2020). Promoting reminiscences with virtual reality headsets: a pilot study with people with dementia. Int J Environ Res Public Health.

[16] Jiang H, Eaglestone G, McCrone P, Carr C, Stoner C (2025). How are nature-based interventions defined in mild cognitive impairment and dementia studies? A conceptual systematic review and novel taxonomy. Dementia (London).

[17] Appel L, Ali S, Narag T (2021). Virtual reality to promote wellbeing in persons with dementia: a scoping review. J Rehabil Assist Technol Eng.

[18] Huygelier H, Schraepen B, van Ee R, Vanden Abeele V, Gillebert CR (2019). Acceptance of immersive head-mounted virtual reality in older adults. Sci Rep.

[19] Strong J (2020). Immersive virtual reality and persons with dementia: a literature review. J Gerontol Soc Work.

[20] Appel L, Kisonas E, Appel E (2021). Administering virtual reality therapy to manage behavioral and psychological symptoms in patients with dementia admitted to an acute care hospital: results of a pilot study. JMIR Form Res.

[21] Kim O, Pang Y, Kim JH (2019). The effectiveness of virtual reality for people with mild cognitive impairment or dementia: a meta-analysis. BMC Psychiatry.

[22] Appel L, Appel E, Kisonas E (2024). Evaluating the impact of virtual reality on the behavioral and psychological symptoms of dementia and quality of life of inpatients with dementia in acute care: randomized controlled trial (VRCT). J Med Internet Res.

[23] Brimelow RE, Dawe B, Dissanayaka N (2020). Preliminary research: virtual reality in residential aged care to reduce apathy and improve mood. Cyberpsychol Behav Soc Netw.

[24] Moyle W, Jones C, Dwan T, Petrovich T (2018). Effectiveness of a virtual reality forest on people with dementia: a mixed methods pilot study. Gerontologist.

[25] Walden A, Feliciano L (2022). A virtual reality intervention to reduce dementia-related agitation using single-case design. Clin Gerontol.

[26] Huang LC, Yang YH (2022). The long-term effects of immersive virtual reality reminiscence in people with dementia: longitudinal observational study. JMIR Serious Games.

[27] Brimelow RE, Thangavelu K, Beattie R, Dissanayaka NN (2022). Feasibility of group-based multiple virtual reality sessions to reduce behavioral and psychological symptoms in persons living in residential aged care. J Am Med Dir Assoc.

[28] Sisco F, Taurel M, Lafont V (2000). Les troubles du comportement chez le sujet dément en institution: évaluation à partir de l’inventaire neuropsychiatrique pour les équipes soignantes (NPI/ES) [Article in French]. L’ Année gérontologique.

[29] Cummings JL, Mega M, Gray K, Rosenberg-Thompson S, Carusi DA, Gornbein J (1994). The Neuropsychiatric Inventory: comprehensive assessment of psychopathology in dementia. Neurology.

[30] Delphin-Combe F, Dauphinot V, Denormandie P (2018). The scale of instantaneous wellbeing: validity in a population with major neurocognitive disorders. Geriatr Psychol Neuropsychiatr Vieil.

[31] Mabire JB, Gay MC, Vrignaud P, Garitte C, Vernooij-Dassen M (2016). Social interactions between people with dementia: pilot evaluation of an observational instrument in a nursing home. Int Psychogeriatr.

[32] Leone E, Lafont V, Filleau C (2008). Validation de la version soignant de l’Inventaire Apathie [Article in French]. Rev Gériatr.

[33] Kubota K, Katayama T, Takamaru K (2026). Immersive virtual reality for older adults with mild cognitive impairment, dementia, or cognitive frailty: a systematic review and narrative synthesis (2019-2025). BMC Geriatr.

[34] Park SM, Choi SY, Kim JH (2022). Effect of virtual reality program for alleviating behavioral and psychological symptoms of dementia patients [Article in Korean]. J Korean Acad Nurs.

[35] Gusdal AK, Gustafsson C (2020). Virtual reality in the care of people with dementia: a single-case research study. J Patient Care Serv.

[36] Kim JH, Park S, Lim H (2021). Developing a virtual reality for people with dementia in nursing homes based on their psychological needs: a feasibility study. BMC Geriatr.

[37] Gueyraud C (2018). Jeu et maladie d'Alzheimer : le jeu libre comme intention de soin en direction de résidents d'EHPAD atteints de la maladie d'Alzheimer ou troubles apparentés [Dissertation] [Article in French]. https://theses.fr/2018LYSE2050.

[38] Travis Seidl JN, Massman PJ (2016). Cognitive and functional correlates of NPI-Q scores and symptom clusters in mildly demented Alzheimer patient. Alzheimer Dis Assoc Disord.

[39] Ishii S, Weintraub N, Mervis JR (2009). Apathy: a common psychiatric syndrome in the elderly. J Am Med Dir Assoc.

[40] Zhao QF, Tan L, Wang HF (2016). The prevalence of neuropsychiatric symptoms in Alzheimer’s disease: systematic review and meta-analysis. J Affect Disord.

[41] Allerhand M, Gale CR, Deary IJ (2014). The dynamic relationship between cognitive function and positive well-being in older people: a prospective study using the English Longitudinal Study of Aging. Psychol Aging.

[42] Llewellyn DJ, Lang IA, Langa KM, Huppert FA (2008). Cognitive function and psychological well-being: findings from a population-based cohort. Age Ageing.

[43] Mabire JB, Gay MC, Vrignaud P, Garitte C, Jeon YH, Vernooij-Dassen M (2018). Effects of active psychosocial stimulation on social interactions of people with dementia living in a nursing home: a comparative study. Int Psychogeriatr.

[44] Lu S, Zhang AY, Liu T (2021). Degree of personalisation in tailored activities and its effect on behavioural and psychological symptoms and quality of life among people with dementia: a systematic review and meta-analysis. BMJ Open.

[45] Wen J, Yan H, Wang S, Xu J, Zhou Z (2024). The effectiveness of nursing interventions for elderly dementia patients based on virtual reality technology: a systematic review and meta-analysis. Ageing Res Rev.

