# Supplementary material for: Effectiveness of a Virtual Reality Immersion Process as a Nonpharmacological Intervention for Behavioral and Psychological Symptoms of Dementia and Immediate Well-Being in People Living in Nursing Homes: Randomized Controlled Trial
Source: JMIR Form Res. 2026 Oct 6;10:e95542. doi: 10.2196/95542 (PMC13641323; doi:10.2196/95542)
Supplement: Supplementary file 2 [file formative-v10-e95542-s001.docx]

## Multimedia Appendix 1. Resident Capacity of Each Centre and Numbers of Participants Enrolled and Included in the Final Analyses.

| Centre – Location | Resident capacity | Number of participants included in the study | Number of participants included in the analyses |
| --- | --- | --- | --- |
| 1 – Pollionnay | 80 | 11 (6 control, 5 intervention) | 11 (6 control, 5 intervention) |
| 2 – Lyon | 103 | 9 (4 control, 5 intervention) | 7 (4 control, 3 intervention) |
| 3 – Lyon | 80 | 12 (6 control, 6 intervention) | 9 (4 control, 5 intervention) |
| 4 – Le Puy-en-Velay | 45 | 12 (6 control, 6 intervention) | 11 (5 control, 6 intervention) |
| 5 – Le Puy-en-Velay | 49 | 12 (6 control, 6 intervention) | 12 (6 control, 6 intervention) |
| Total |  | 56 (28 control, 28 intervention) | 50 (25 control, 25 intervention) |
