# Supplementary material for: Effectiveness of a Virtual Reality Immersion Process as a Nonpharmacological Intervention for Behavioral and Psychological Symptoms of Dementia and Immediate Well-Being in People Living in Nursing Homes: Randomized Controlled Trial
Source: JMIR Form Res. 2026 Oct 6;10:e95542. doi: 10.2196/95542 (PMC13641323; doi:10.2196/95542)
Supplement: Supplementary file 4 [file formative-v10-e95542-s003.docx]

## Multimedia Appendix 3. Distribution of patients for each NPI symptom by group (intervention/control) and study phase (pre-/post-intervention), according to frequency and severity levels

| **Symptom** | **Phase** | **Group** | **N/n** | **Frequency** | | | | **Severity** | | |
| --- | --- | --- | --- | --- | --- | --- | --- | --- | --- | --- |
|  |  |  |  | **1** | **2** | **3** | **4** | **1** | **2** | **3** |
| Delusions | Pre | Total | 16 | 0 | 4 | 8 | 4 | 4 | 9 | 3 |
|  |  | Intervention | 6 | 0 | 1 | 4 | 1 | 1 | 3 | 2 |
|  |  | Control | 10 | 0 | 3 | 4 | 3 | 3 | 6 | 1 |
|  | Post | Total | 15 | 0 | 4 | 6 | 5 | 3 | 8 | 3 |
|  |  | Intervention | 6 | 0 | 1 | 3 | 2 | 1 | 3 | 1 |
|  |  | Control | 9 | 0 | 3 | 3 | 3 | 2 | 5 | 2 |
| Hallucinations | Pre | Total | 4 | 2 | 2 | 0 | 0 | 3 | 0 | 1 |
|  |  | Intervention | 2 | 1 | 1 | 0 | 0 | 2 | 0 | 0 |
|  |  | Control | 2 | 1 | 1 | 0 | 0 | 1 | 1 | 0 |
|  | Post | Total | 3 | 3 | 0 | 0 | 0 | 2 | 1 | 0 |
|  |  | Intervention | 0 | 0 | 0 | 0 | 0 | 0 | 0 | 0 |
|  |  | Control | 3 | 3 | 0 | 0 | 0 | 2 | 1 | 0 |
| Agitation | Pre | Total | 25 | 4 | 6 | 7 | 8 | 5 | 11 | 9 |
|  |  | Intervention | 11 | 1 | 4 | 2 | 4 | 0 | 5 | 6 |
|  |  | Control | 14 | 3 | 2 | 5 | 4 | 5 | 6 | 3 |
|  |  | Total | 26 | 0 | 11 | 10 | 5 | 8 | 12 | 6 |
|  | Post | Intervention | 12 | 0 | 5 | 5 | 2 | 2 | 7 | 3 |
|  |  | Control | 14 | 0 | 6 | 5 | 3 | 6 | 5 | 3 |
| Depression/Dysphoria | Pre | Total | 35 | 3 | 5 | 13 | 14 | 10 | 17 | 8 |
|  |  | Intervention | 17 | 1 | 2 | 6 | 8 | 4 | 8 | 5 |
|  |  | Control | 18 | 2 | 3 | 7 | 6 | 6 | 9 | 3 |
|  | Post | Total | 34 | 3 | 7 | 14 | 10 | 11 | 18 | 5 |
|  |  | Intervention | 16 | 0 | 5 | 5 | 6 | 5 | 8 | 3 |
|  |  | Control | 18 | 3 | 2 | 9 | 4 | 6 | 10 | 2 |
| Anxiety | Pre | Total | 35 | 4 | 5 | 11 | 15 | 8 | 9 | 18 |
|  |  | Intervention | 14 | 1 | 4 | 2 | 7 | 2 | 5 | 7 |
|  |  | Control | 21 | 3 | 1 | 9 | 8 | 6 | 4 | 11 |
|  | Post | Total | 35 | 6 | 3 | 12 | 14 | 10 | 14 | 11 |
|  |  | Intervention | 15 | 2 | 2 | 4 | 7 | 4 | 8 | 3 |
|  |  | Control | 20 | 4 | 1 | 8 | 7 | 6 | 6 | 8 |
| Euphoria | Pre | Total | 5 | 1 | 2 | 0 | 2 | 2 | 3 | 0 |
|  |  | Intervention | 3 | 1 | 0 | 0 | 2 | 1 | 2 | 0 |
|  |  | Control | 2 | 0 | 2 | 0 | 0 | 1 | 1 | 0 |
|  | Post | Total | 6 | 2 | 2 | 1 | 1 | 3 | 3 | 0 |
|  |  | Intervention | 3 | 1 | 1 | 0 | 1 | 1 | 2 | 0 |
|  |  | Control | 3 | 1 | 1 | 1 | 0 | 2 | 1 | 0 |
| Apathy | Pre | Total | 29 | 1 | 2 | 10 | 16 | 2 | 8 | 19 |
|  |  | Intervention | 15 | 0 | 1 | 3 | 11 | 1 | 3 | 11 |
|  |  | Control | 14 | 1 | 1 | 7 | 5 | 1 | 5 | 8 |
|  | Post | Total | 31 | 3 | 1 | 12 | 15 | 4 | 13 | 14 |
|  |  | Intervention | 17 | 1 | 0 | 5 | 11 | 2 | 6 | 9 |
|  |  | Control | 14 | 2 | 1 | 7 | 4 | 2 | 7 | 5 |
| Desinhibition | Pre | Total | 8 | 0 | 4 | 2 | 2 | 3 | 4 | 1 |
|  |  | Intervention | 4 | 0 | 2 | 0 | 2 | 1 | 2 | 1 |
|  |  | Control | 4 | 0 | 2 | 2 | 0 | 2 | 2 | 0 |
|  | Post | Total | 9 | 1 | 3 | 2 | 3 | 4 | 4 | 1 |
|  |  | Intervention | 4 | 1 | 0 | 1 | 2 | 2 | 2 | 0 |
|  |  | Control | 5 | 0 | 3 | 1 | 1 | 2 | 2 | 1 |
| Irritability | Pre | Total | 17 | 3 | 6 | 2 | 6 | 5 | 9 | 3 |
|  |  | Intervention | 9 | 2 | 3 | 2 | 2 | 3 | 4 | 2 |
|  |  | Control | 8 | 1 | 3 | 0 | 4 | 2 | 5 | 1 |
|  | Post | Total | 18 | 3 | 7 | 2 | 6 | 7 | 11 | 0 |
|  |  | Intervention | 10 | 2 | 4 | 2 | 2 | 5 | 5 | 0 |
|  |  | Control | 8 | 1 | 3 | 0 | 4 | 2 | 6 | 0 |
| Aberrant motor activity | Pre | Total | 19 | 2 | 2 | 4 | 11 | 5 | 7 | 7 |
|  |  | Intervention | 8 | 1 | 1 | 1 | 5 | 2 | 4 | 2 |
|  |  | Control | 11 | 1 | 1 | 3 | 6 | 3 | 3 | 5 |
|  | Post | Total | 18 | 1 | 3 | 5 | 9 | 9 | 6 | 3 |
|  |  | Intervention | 8 | 0 | 2 | 2 | 4 | 6 | 1 | 1 |
|  |  | Control | 10 | 1 | 1 | 3 | 5 | 3 | 5 | 2 |
| Night-time behaviour | Pre | Total | 11 | 2 | 1 | 6 | 2 | 5 | 4 | 2 |
|  |  | Intervention | 5 | 1 | 1 | 3 | 0 | 3 | 1 | 1 |
|  |  | Control | 6 | 1 | 0 | 3 | 2 | 2 | 3 | 1 |
|  | Post | Total | 12 | 2 | 2 | 6 | 2 | 7 | 4 | 1 |
|  |  | Intervention | 5 | 0 | 2 | 3 | 0 | 3 | 1 | 1 |
|  |  | Control | 7 | 2 | 0 | 3 | 2 | 4 | 3 | 0 |
| Appetite and eating change | Pre | Total | 8 | 2 | 2 | 0 | 4 | 3 | 5 | 0 |
|  |  | Intervention | 3 | 1 | 1 | 0 | 1 | 2 | 1 | 0 |
|  |  | Control | 5 | 1 | 1 | 0 | 3 | 1 | 4 | 0 |
|  | Post | Total | 8 | 2 | 2 | 1 | 3 | 3 | 4 | 1 |
|  |  | Intervention | 3 | 1 | 1 | 0 | 1 | 2 | 1 | 0 |
|  |  | Control | 5 | 1 | 1 | 1 | 2 | 1 | 3 | 1 |
