# Supplementary material for: Effectiveness of a Virtual Reality Immersion Process as a Nonpharmacological Intervention for Behavioral and Psychological Symptoms of Dementia and Immediate Well-Being in People Living in Nursing Homes: Randomized Controlled Trial
Source: JMIR Form Res. 2026 Oct 6;10:e95542. doi: 10.2196/95542 (PMC13641323; doi:10.2196/95542)
Supplement: Supplementary file 5 [file formative-v10-e95542-s004.docx]

## Multimedia Appendix 4. Attendance and reasons for not attending for each session.

| Session | Attendance to the session | | Reason for not attending the session | |
| --- | --- | --- | --- | --- |
|  |  |  |  |  |
|  | Yes | No |  |  |
| Randomisation | 56 | 0 | Refusal | 0 |
|  |  |  | Medical reason | 0 |
|  |  |  | Other | 0 |
| 1 | 43 | 11 | Refusal | 2 |
|  |  |  | Medical reason | 4 |
|  |  |  | Other | 5 |
| 2 | 42 | 12 | Refusal | 7 |
|  |  |  | Medical reason | 4 |
|  |  |  | Other | 1 |
| 3 | 43 | 11 | Refusal | 1 |
|  |  |  | Medical reason | 6 |
|  |  |  | Other | 4 |
| 4 | 44 | 10 | Refusal | 2 |
|  |  |  | Medical reason | 5 |
|  |  |  | Other | 3 |
| 5 | 45 | 9 | Refusal | 3 |
|  |  |  | Medical reason | 5 |
|  |  |  | Other | 1 |
| 6 | 35 | 19 | Refusal | 8 |
|  |  |  | Medical reason | 8 |
|  |  |  | Other | 3 |
| 7 | 38 | 14 | Refusal | 5 |
|  |  |  | Medical reason | 6 |
|  |  |  | Other | 3 |
| 8 | 42 | 8 | Refusal | 3 |
|  |  |  | Medical reason | 4 |
|  |  |  | Other | 1 |
| 9 | 42 | 8 | Refusal | 5 |
|  |  |  | Medical reason | 1 |
|  |  |  | Other | 2 |
| 10 | 40 | 10 | Refusal | 5 |
|  |  |  | Medical reason | 1 |
|  |  |  | Other | 4 |
| 11 | 33 | 17 | Refusal | 7 |
|  |  |  | Medical reason | 3 |
|  |  |  | Other | 7 |
| 12 | 34 | 14 | Refusal | 7 |
|  |  |  | Medical reason | 3 |
|  |  |  | Other | 4 |
