# Supplementary material for: Effectiveness of a Virtual Reality Immersion Process as a Nonpharmacological Intervention for Behavioral and Psychological Symptoms of Dementia and Immediate Well-Being in People Living in Nursing Homes: Randomized Controlled Trial
Source: JMIR Form Res. 2026 Oct 6;10:e95542. doi: 10.2196/95542 (PMC13641323; doi:10.2196/95542)
Supplement: Supplementary file 6 [file formative-v10-e95542-s002.docx]

# Multimedia Appendix 2: Immediate Well-Being Evaluation (IWBE)
